# Genetically Encoded *Levivirus* Coat Protein-Based Yeast Display Libraries of Cyclic Peptides

**DOI:** 10.1021/acssynbio.4c00873

**Published:** 2025-07-14

**Authors:** Theodor Simak, Florian Stracke, Oskar Smrzka, Gordana Wozniak-Knopp

**Affiliations:** † Christian Doppler Laboratory for Innovative Immunotherapeutics, BOKU University, Muthgasse 18, 1190 Vienna, Austria; ‡ Ablevia biotech GmbH, Maria Jacobi Gasse 1, 1030 Vienna, Austria

**Keywords:** antibody−antigen interaction, autoantibodies, cyclic peptides, epitope mapping, neurological disease, yeast display

## Abstract

Peptide libraries present have improved means to effectively identify epitopes of antibodies, either as monoclonal reagents or polyclonal constituents of the immune response, which includes characterization of vaccination responses, profiling of allergic reactions, and screening of patient samples for autoantibodies. In all of these examples, there is an urgent demand for simple and inexpensive target epitope screening. Here, we present a method for epitope identification, based on the yeast display of overlapping peptides conformationally constrained within the *Levivirus* capsid protein PP7 with the aid of a disulfide bridge. Using rituximab as a model antibody, the PP7 scaffold was screened for favorable positions for the grafting of peptides, which should allow high accessibility and efficient cyclization. Libraries of overlapping peptide fragments were then constructed, affinity-selected, and screened to retrieve the correct epitopes of model monoclonal antibodies through the enrichment of affinity-captured sequences. Further, plasma rich in antiglutamic acid decarboxylase (GAD) 65 antibodies, a phenomenon associated with a number of neurological disorders, such as “stiff-person-syndrome”, was successfully used as a bait to discover the relevant epitope from the antigen peptide library. The presented system sets the basis for a platform that could contribute to novel diagnostic approaches and the discovery of antigen-specific treatments, conducive to a precision medicine approach superior to generalized immunosuppression.

## Graphical abstract

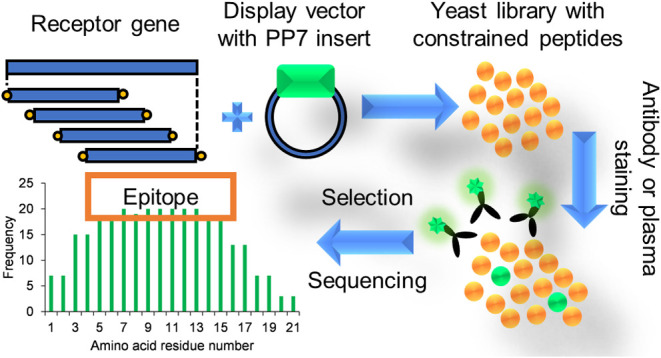

## Introduction

Peptide libraries have been an invaluable tool for the identification of biologically important ligands to several different targets, most notably diverse receptors, enzymes, and antibodies.^1,2^ Both chemically produced and genetically encoded libraries have been a productive source for the identification of sequences informative in various applications, including mapping of binding sites or epitopes and design of ligands mimicking the activity of natural binding partners.^3^ Several reports declare that the discovery of peptide-binding motifs has been more efficient when based on cyclized sequences than with linear ones, which is due to additional structural homogeneity introduced by the constraint,^4,5^ as well as an increase in the surface area available for interaction with the biological target, both of which lead to a higher binding affinity.^6^ This has been observed several times for chemically produced libraries as well as for peptides selected *in vivo*, for example, via panning of the phage display library.^7^ A second important advantage of the cyclic peptides is their lower propensity for proteolysis.^8^ Chemical synthesis of cyclic peptides has been efficiently achieved using several methods, including cyclization in solution using coupling agents, native chemical ligation, various chemoselective cyclizations, on-resin cyclization, and enzyme-mediated cyclization (recently reviewed in^7^). In genetically encoded libraries, which are more readily constructed and often more easily sourced than their chemical counterparts, the spontaneous cyclization of peptide sequences *in vivo* is only partially efficient when simple addition of a single cysteine residue at each N- and C-terminal side of the encoded peptide is used.^9^ To overcome the potential heterogeneity in the expressed peptide construct, several ingenious solutions have been proposed. For the phage platform, cyclization of phage-produced peptides can be controlled to form two distinct loops after chemical modification with tris­(bromomethyl)­benzene that facilitates the pairing of reactive cysteines.^9^ For the bacterial display system, split-intein circular ligation of peptides and proteins (SICLOPPS) was designed for the intracellular production of cyclic peptides and was successfully used to identify inhibitory ligands for enzymes and those interfering with the protein–protein interaction.^10^ Sophisticated strategies embodied in the yeast display system describe the cyclization of the expressed linear peptide, achieved with post-translational modification with site-selective enzymatic treatment with bacterial transglutaminase,^11^ or use cyclization via click chemistry following the incorporation of an unnatural amino acid into the displayed peptide sequence.^12^

The yeast display platform has since decades been very popular as a straightforward method for the selection of bait-specific binding partners, employing fluorescence-activated cell selection (FACS) for the enrichment of relevant clones.^13^ The yeast display has been instrumental to epitope mapping since more than 20 years.^14–16^ Recent notable examples include the display of the antigen mutant library, the subsequent selection with antibodies for variants showing a decrease in antibody binding, the evaluation of their frequency in comparison with the original library, and the structural mapping of enriched positions for epitope determination.^17,18^ Since it became simple to generate large pools of DNA oligos, yeast libraries expressing millions of small overlapping peptides were employed to identify linear epitopes with high resolution, up to a single amino acid residue.^19^ To leverage the advantage of superior antibody recognition of cyclicized peptides, we aimed at efficiently displaying cyclic peptide stretches on the surface of yeast and employed the *Levivirus* phage protein PP7 capsid protein as a scaffold for this purpose. In its naturally occurring dimer, the protomers are connected with a β-hairpin motif, and this site readily accommodates insertions in variable sizes reaching up to an entire antibody single domain.^20^ Aside from this conventional insertion site, we also explored other positions in the PP7 capsid protein that could alternatively or in addition support the presentation and cyclization of peptides via N-and C-terminal cysteine pairing. The efficiency of cyclization was first assessed using a model peptide pertaining to the CD20 extracellular domain, recognized by the clinically validated antibody rituximab (RX).^21^ A further model, the cetuximab (CX) meditope peptide, whose activity critically depends on its cyclization,^22^ was used as a target for binding of its cognate antibody. The amenability of the novel insertion sites for linear peptide recognition was also evaluated, using the hemagglutinin (HA) recognition motif^23^ and an anti-HA antibody as a detecting agent. Once the best insertion site was identified, the highly displayed PP7 protein was used as an acceptor for genetically encoded constrained peptide libraries.

Further, we were interested to examine the potential of this novel platform to deliver peptide hits to certain antibodies enriched in human plasma, which would pave the way for several applications of the proposed system, such as delineation of target epitopes of the immune response toward infection or vaccines. On the other hand, serum-enriched pathogenic antibodies are causative agents of several types of autoimmune diseases, as discovered, for example, myasthenia gravis,^24^ anti-*N*-methyl-d-aspartate receptor (NMDAR) encephalitis,^25^ and neuromyelitis optica.^26^ Their targets are typically multidomain membrane proteins that are difficult to express and isolate in high quantities,^27^ and cyclic peptides representing or mimicking their activity are valuable surrogate tools for the diagnosis, prediction, and monitoring of autoimmune diseases. Provided sufficient specificity, they ultimately present a basis for the design of ligands that can be used for selective depletion of such pathogenic antibodies. Among several other types of autoantibodies established as pathogenic agents for autoimmune diseases, glutamic acid decarboxylase 65 kDa isoform (GAD65)-directed antibodies have been connected with diabetes mellitus type 1 ^28,29^ and diverse neurological disorders, such as stiff-person syndrome, cerebellar ataxia, limbic encephalitis, and epilepsy.^30^ To discover their relevant target peptide motifs from a yeast-PP7 capsid protein platform, we sorted the yeast display library containing GAD65-derived cyclized peptides to identify those reactive with an anti-GAD65-positive plasma. Finally, we aimed at defining the potential limitations of our “phage-on-yeast” method by testing the background reactivity of 50 plasma samples with yeast cells not displaying any peptides and designed a protocol for the depletion of interfering factors skewing the read-out.

## Results and Discussion

We designed a novel approach for the identification of cyclic peptides as antibody epitopes using the yeast display system, which combines simple library construction, efficient monitoring of the selection outcome via FACS-assisted protocols, and the possibility of normalization of response to the bait to the display level for every cell participating in library selection (the scheme of the display construct is displayed in Figure 1A and the scheme of the genetic construct is displayed in Figure S1). Efficient cyclization should be achieved through grafting of the peptide at an appropriate site of the PP7 phage coat protein.

**Figure 1:**
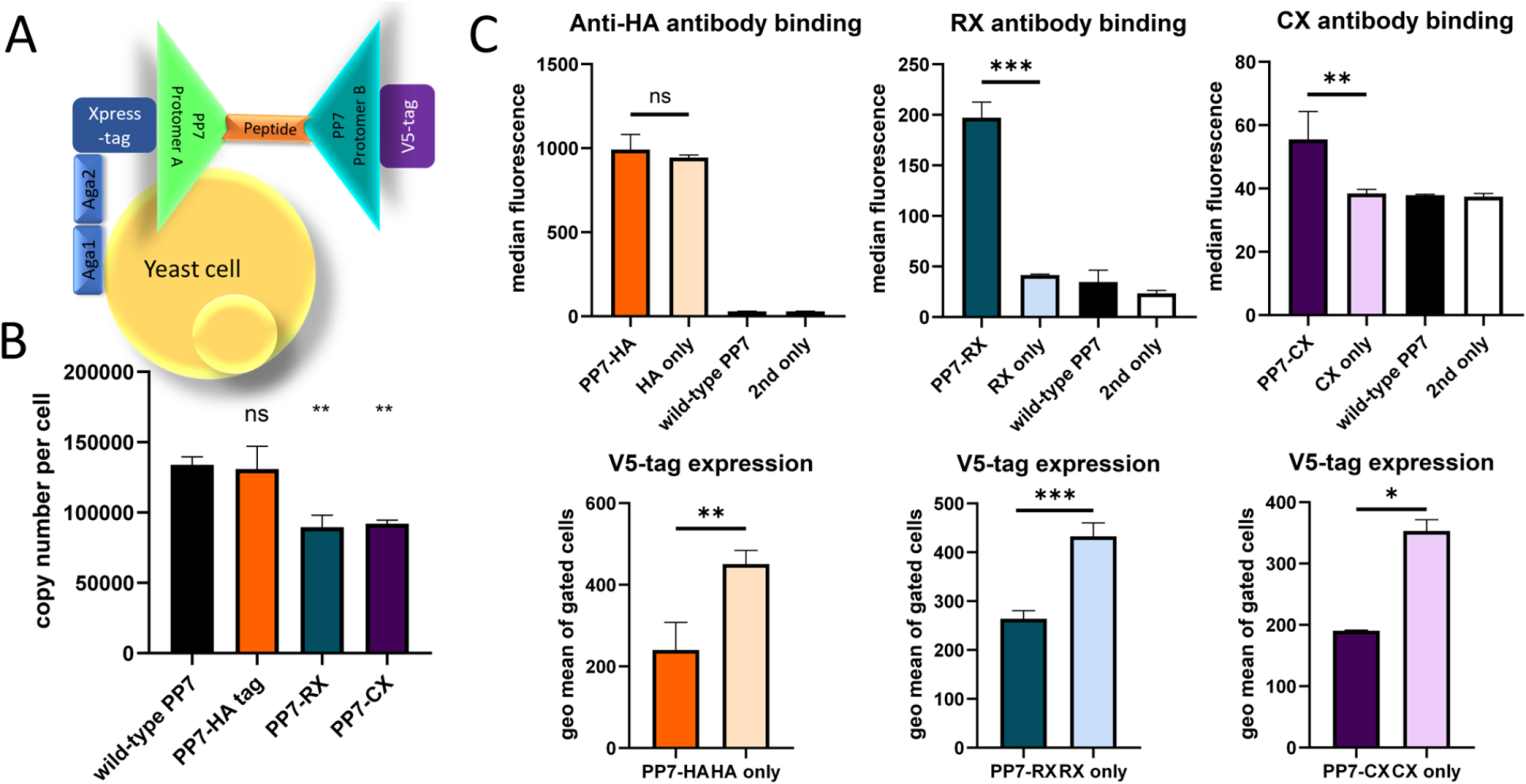
Yeast display of the phage PP7 capsid protein with grafted peptides. (A) Design of the expression construct with the N-terminal Xpress-tag, PP7 protomer A, inserted peptide, PP7 protomer B, and C-terminal V5-tag. (B) Copy number of the displayed construct (middle “MI” position) determined using QIFIkit for calibration. Mean values and standard deviations as error bars are shown. The significance was calculated with one-way ANOVA in comparison with wild-type PP7 (ns: not significant, *P* > 0.05, **: 0.01 < *P* < 0.005). (C) Specific antibody binding (upper panels) and the display of constructs determined via the C-terminal V5-tag (lower panels). Peptide-grafted PP7 are compared with the peptide displayed without the PP7 scaffold (ns: not significant, *P* > 0.05, *: 0.05 < *P* < 0.01, **: 0.01 < *P* < 0.005, ***: *P* < 0.005; 2nd only: secondary reagent only).

### Display of the PP7 Protein and Grafted Constrained Peptides

The yeast display of the PP7 capsid protein was determined to be at 140,000 copies per cell, which is considered highly efficient for the yeast system.^31^ In the initial experiment, the HA-tag, the CD20 peptide with the binding site for RX (RX-peptide), and the CX-meditope peptide were introduced to the conventional insertion site between protomers, here designated as “MI” for “middle”, to estimate the potential for cyclization, as well as their accessibility. The display of the peptides was efficient with all three constructs and did not significantly differ from the wild-type PP7 for the HA-tag, while it was lower, at about 100,000 copies per cell, for RX- and CX-cognate peptides (Figure 1B). When the peptides were displayed without the PP7 scaffold, the reactivity of the anti-HA antibody was not significantly different from PP7 protein-incorporated antigen, while RX and CX did not react with the peptides without the PP7 scaffold, although they were displayed at a higher level (Figure 1C).

Next, we examined the effect of terminal cysteine residues encompassing the peptide positioned between the PP7 capsid protein protomers to discover if they indeed support the cyclic constraint of the peptide. When they were mutated to alanine, there was significantly less reactivity of the CX antibody with CX-meditope (Figure 2A), and for RX, the binding was reduced, at a comparable or a higher display level (Figure 2B). At this point, we were also interested in how exactly the pairing of cysteine residues in the displayed RX-cognate peptide proceeds, as there is a single cysteine present in the peptide sequence itself. We therefore compared mutants with the changes of one single, two, or all three cysteines to alanine (C1A, C7A and C18A) (Figure 2B and representative dot plots shown in Figure S2). Surprisingly, the C7A mutant was well expressed and reactive with RX, so we have chosen this one for the future experiments that would investigate further options of cysteine-mediated cyclization in the PP7 capsid protein scaffold, as here we could exclude the pairing of C7 cysteine to any of the neighboring ones, which adheres more consistently to the concept of the peptide graft between two cysteine residues.

**Figure 2:**
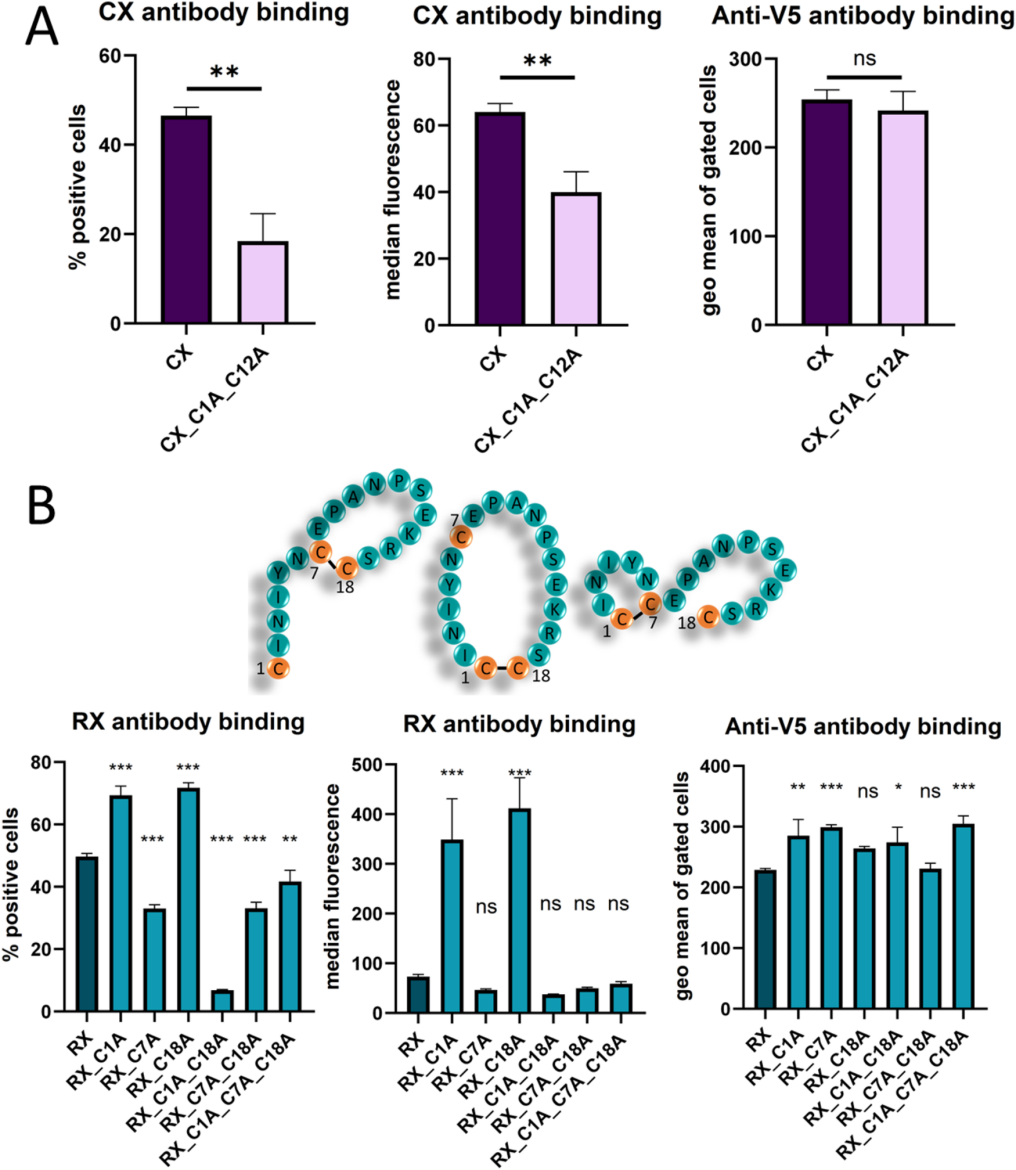
Influence of N- and C-terminal cysteine residues on the peptide constraint when inserted between the protomers (at the MI position) in PP7. (A) CX-meditope peptide binding of the CX antibody and the level of display determined via C-terminal V5-tag reactivity. (B) RX-peptide binding of the RX antibody and the level of display determined via C-terminal V5-tag reactivity; additionally, the influence of C7A (the inherent cysteine residue in the peptide sequence as presented in the string-of-pearls diagram) was studied. The cognate antibody binding and the level of display were compared with the original peptide graft within the PP7 scaffold with one-way ANOVA (ns: not significant, *P* > 0.05, *: 0.05 < *P* < 0.01, **: 0.01 < *P* < 0.005, ***: *P* < 0.005). In the used yeast display system, a part of the yeast population (20–30%) is always completely negative for expression, which is why we present the geometric mean of positive cells for well-separated populations after anti-V5 antibody staining and percent positive cells or the median fluorescence intensity or both for other stainings.

To this end, apart from the middle “MI” position between the protomers, we tried to discover others that could be utilized for cysteine-mediated cyclization and accommodation of the peptides. We have applied the MODIP algorithm^32^ of DSD-BASE^33^ to search for pairs of amino acids that can be exchanged for a pair of cysteines in the PP7 dimer structure PDB: 2QUD.^34^ The suggested amino acid residues were then inspected for their position in the PP7 capsid protein structure and the orientation of their side chains in the original PDB, but also for surface exposure in PDB: 6N4V,^20^ which is a cryoelectron microscopy-derived structure of the multimeric PP7 capsid protein. Two positions in protomer A and three positions in protomer B were selected for modification (Figure 3A). The RX-cognate peptide sequence, once as a wild-type and once containing the C7A mutation, were introduced, and the reactivity with the RX antibody and anti-HA antibody was determined (Figure 3B). The level of display was compared for these variants using the binding of the anti-V5 antibody to the C-terminal V5-tag. Judging from the reactivity with the HA antibody, all proposed novel positions were reactive to a different degree; however, A1 appeared most favorable for antibody binding. The reactivity of the RX-cognate peptide with RX could be measured when incorporated at positions A1 and B1, and the RX-C7A variant was additionally positive for binding at position B4. We were interested if the CX-meditope can be introduced at position A1, but we could not measure any reactivity with the CX antibody for that construct (Figure 3B). While position A1 was more favorable than MI for the display of RX-C7A, the other two model peptides were better reactive when positioned at MI, and the same was true for the HA-tag.

**Figure 3:**
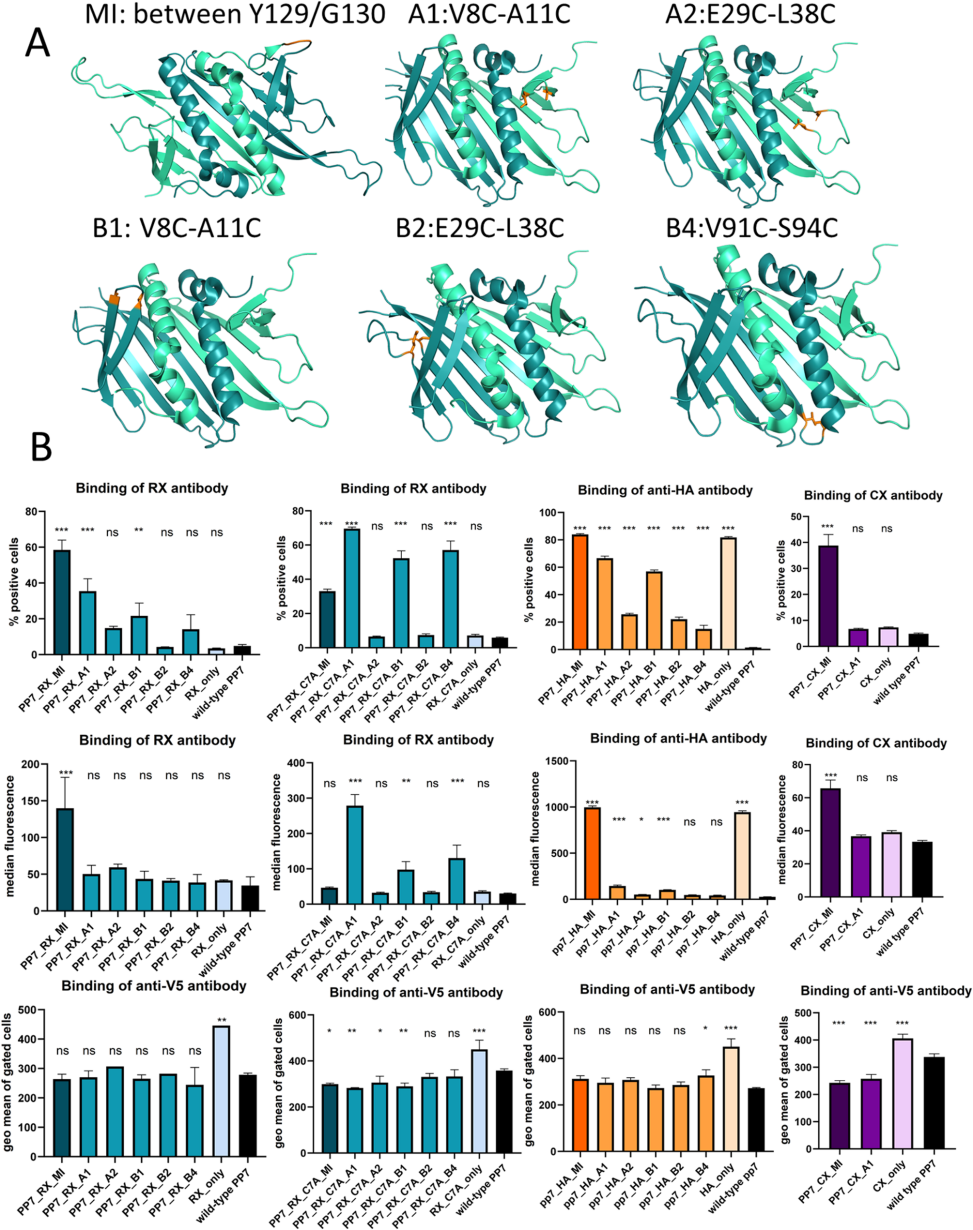
Exploring the PP7 scaffold for potential cysteine pairs that could accommodate the constrained peptide graft. (A) Cartoon diagrams of proposed variants of the PP7 capsid protein (PDB ID: 2QUD; protomer A: pale green; protomer B: teal; residues modified to cysteine encompassing the grafted peptide sequence: orange). “MI” indicates the positions between the protomers (“middle”). The figure was prepared using PyMOL version 2.4.0. (B) Binding of cognate RX, anti-HA, and CX antibodies to the yeast displaying PP7 grafted with RX, RX_C7A, or HA insert or the insets only (upper two rows) and the level of display for those determined via C-terminal V5-tag reactivity, in comparison with wild-type PP7 (lower row). The level of significance was determined with one-way ANOVA (ns: not significant, *P* > 0.05, *: 0.05 < *P* < 0.01, **: 0.01 < *P* < 0.005, ***: *P* < 0.005).

### Peptide Libraries for Antibody Epitope Mapping

With that, we decided to produce libraries of overlapping 15-meric peptides with the offset of 2 amino acids, tethered between two cysteines in the MI position (sequences of source antigen sequences in Supporting Information Table S1). A pilot yeast transformation experiment was performed to optimize the mass ratio of the inset to the linearized vector to 40:1. Yeast libraries were constructed at the sizes of 12,200 independent members for influenza HA and 13,500 for CD20. They contained about 90% clones without frameshifts and mutations; the only feature deviating from the design was the number of amino acids in the inset, which was 15 in about 90% of the clones but ranged from 11 to 21. The phenotypic quality control of the libraries was performed, and we determined a high degree of expression of N-terminal Xpress-tag and C-terminal V5-tag, confirming a high percentage of clones with the correct reading frame (Figure 4A). In the unsorted HA-library, 4% of the clones were stained with the anti-HA antibody, while the percentage of RX-binding clones in the CD20-library was lower, about 1% over the background of wild-type PP7 (Figures 4A, S3 and S4). In a single sorting round, the binding clones were significantly enriched. Clones binding to the anti-HA antibody were isolated; 20 were sequenced, and their sequences aligned well with the reported epitope of the anti-HA antibody (Figure 4B). Similar results were achieved for RX, with 44 sequenced clones (Figure 4B). At this point, we investigated if the distribution of amino acid hits will be different if the specific fluorescent signal resulting from bait antibody binding, normalized for the fluorescent signal of the expression reporter antibody binding to the V5-tag, is used as a read-out. In our case, the outcome was the same as when only the frequency of the positive clones was considered, likely because there were no large discrepancies in the V5 signals of the examined clones. This finding is important as the variability of the display level in the phage system, which does not offer an inherent possibility of normalization in contrast to yeast, is known to influence and bias the outcome of the selection.^35^

**Figure 4:**
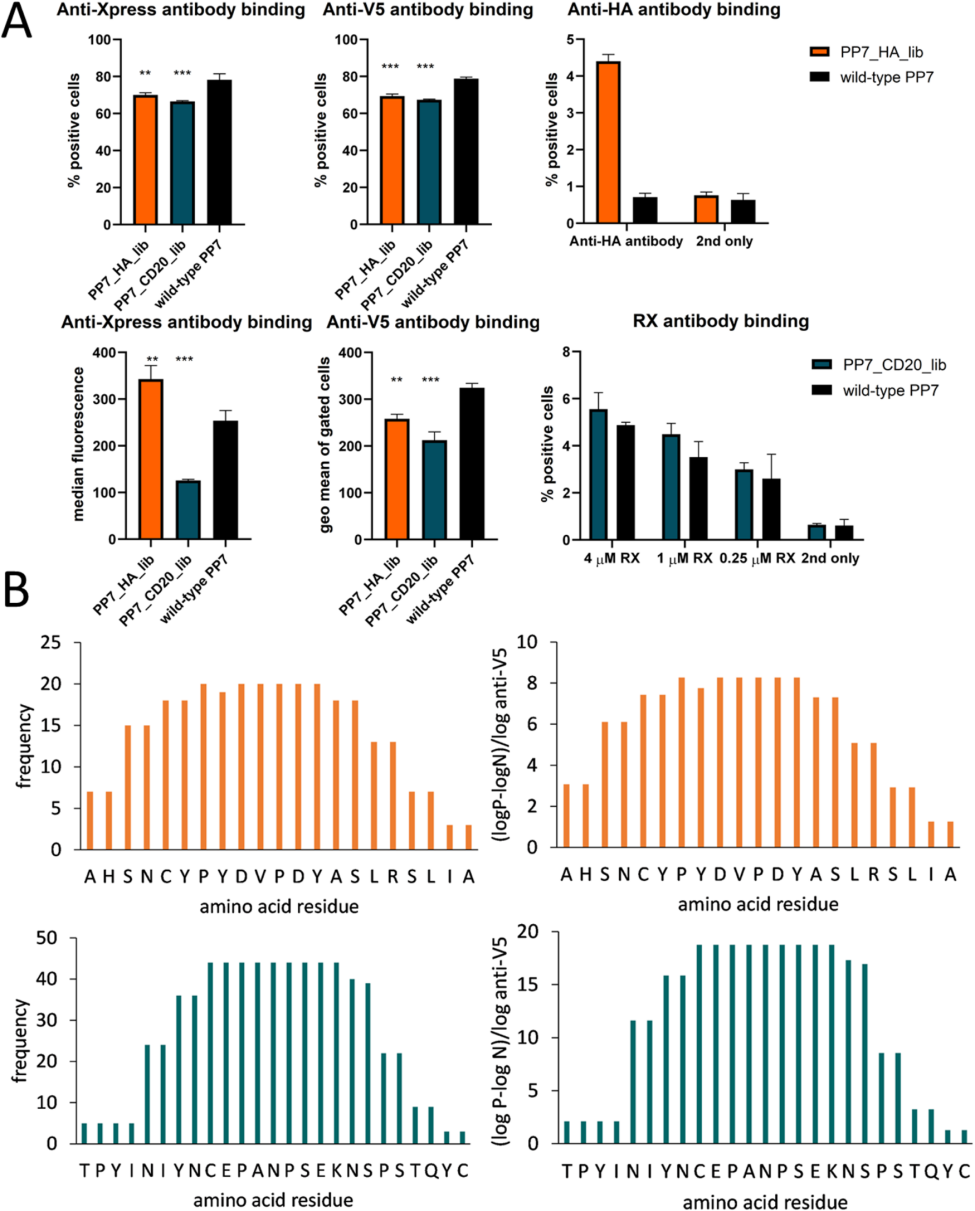
Epitope mapping of the anti-HA and RX antibodies. (A) Libraries of overlapping 15-meric peptides of influenza hemagglutinin and CD20 characterized for the presence of N- and C-terminal tags and the binding of the cognate antibody prior to selection, compared with the yeast clone displaying wild-type PP7 using one-way ANOVA (**: 0.01 < *P* < 0.005, ***: *P* < 0.005). (B) Single yeast clones that reacted with the cognate antibodies anti-HA (upper) and RX (lower) were sequenced, and the sequences were aligned to reconstruct the relevant epitopes; the number of occurrences of an amino acid residue was either only summed by the frequency of the clone (left) or weighted by assigning a factor of specific fluorescence normalized by the expression level (right) (2nd only: secondary reagent only).

### Discovery of Epitopes of Polyclonal Anti-GAD65 Antibodies in the Plasma Sample

We were interested in applying the PP7-assisted display of peptides for the identification of epitopes of plasma antibodies, whose enrichment may present a pathologic condition. The yeast display library expressing the GAD65 sequence in 15-meric peptides with a 14-amino-acid overlap was constructed at a size of 10.000 independent members and sorted with anti-GAD65-positive plasma, diluted 1:20 (Figure 5A). In parallel, the library was stained with an unrelated plasma sample originated from a healthy donor, to exclude the fact that the observed enrichment was artifactual. In the first sorting round, the upper 0.2% of double-positive events were sorted and propagated, and in the second sorting round, the gate was set to double-positive cells falling into the gate of 0.1% false double-positive events stained with the unrelated plasma, and their percentage was 0.8%. Sorted cells were then screened as isolated clones using a polyclonal antibody preparation isolated from the same patient’s plasma sample (Figure 5B). Out of 22 clones, 8 tested positive and were then sequenced. Three different peptides were isolated, encompassing the same epitope (Figure 5C). The discovered sequences were mapped to the GAD65 amino acid residues 509–527, and most of them contained the stretch 513–525 (numbering as in PDB: 2OKK^36^) (Figure 5D). This region contains a large cluster of charged amino acid residues and is located in the small domain of GAD65, composed of helical elements and partially disordered, so its precise crystal structure has not been determined yet.^37^ Interestingly, using mutagenesis studies, this segment has already been identified as an epitope of several human anti-GAD65 monoclonal antibodies, derived from human patients.^38^ Notably, this is also the region with the least homology with GAD67 ^38^ and shares little similarity with other metazoan GAD65 variants as well as with Gad A from , so in this case, molecular mimicry cannot be proposed as a likely factor for autoantibody development.^37^ To conclude, our data provide a starting point for further exploring the structural and functional relevance of this anti-GAD65 epitope.

**Figure 5:**
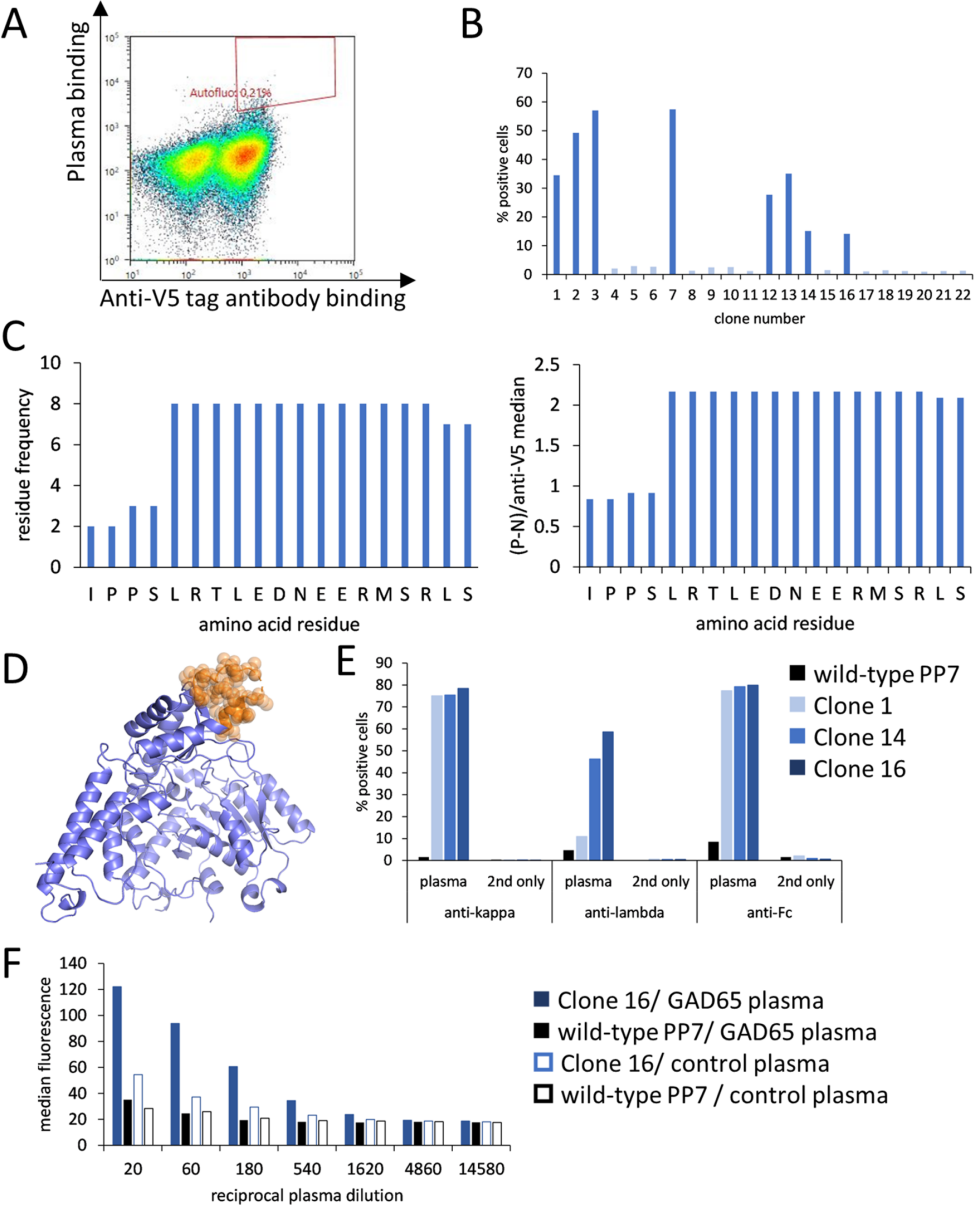
Screening reactive epitopes of autoantibodies in anti-GAD65-positive plasma. (A) GAD65-based peptide library stained with anti-GAD65 plasma and anti-V5 antibody prior to sorting with the indicated sorting gate. (B) Reactivity of isolated clones with polyclonal IgG preparation from the same plasma. (C) Aligned sequences of positive clones; the number of occurrences of an amino acid residue was either only summed by the frequency of the clone (left) or weighted by assigning a factor of specific fluorescence normalized by the expression level (right). (D) Cartoon presentation of GAD65 (PDB: 2OKK) with the sequence of the peptide hit in orange spheres; the figure was prepared with PyMOL version 2.4.0. (E) Discovered yeast clones stained with anti-GAD65 plasma and detected with antikappa, antilambda, or anti-Fc secondary antibodies. (F) A positive yeast clone stained with a dilution series of anti-GAD65-positive plasma and a control plasma.

The three yeast clones harboring different GAD65 peptide sequences were then stained again with the polyclonal antibody preparation, and the binding was detected with different detection reagents: antikappa, antilambda, and anti-Fc antibodies (Figure 5E). While the best response was achieved with anti-Fc antibodies, antilambda staining was present for two yeast display clones, but the response revealed with antikappa was stronger; so we can conclude that the relevant antibodies were mostly of the kappa isotype. When the GAD65-positive plasma sample was used for staining together with the anti-Fc serum, the specific response of the strongest binding clone could be detected down to 1:540 dilution, and the response to the unrelated plasma sample measured in parallel was lower at all dilutions tested (Figure 5F).

### Variable Background Reactivity of Plasma Samples

When determining the background binding to the wild-type PP7 scaffold, we noticed the variable reactivity of the GAD65-positive and an unrelated plasma sample used for monitoring of specificity during the sorting campaign. We therefore expanded the testing of background binding to yeast cells, the empty vector expressing only anti-Xpress, c-myc-, and his-tag, and the wild-type PP7 capsid protein expressed in this context, to further 50 plasma samples of healthy blood donors (Figure 6A). All samples producing a signal significantly higher than the background were positive also when incubated with yeast cells alone, so we concluded that the antiyeast antibodies are the source of this undesired reaction. This information is relevant as the prevalence of anti-*Saccharomyces* antibodies is reported to be relatively high, around 2.8%, in healthy population^39^ and their high levels are a recognized marker for Crohn’s disease^40,41^ as well as other autoimmune conditions.^42^ To deplete those, plasma was incubated with the yeast cell suspension and then clarified by centrifugation and filtration, and the reactivity of the plasma preparation processed in this way decreased to the background level when retested for binding to yeast cells (Figure 6B).

**Figure 6:**
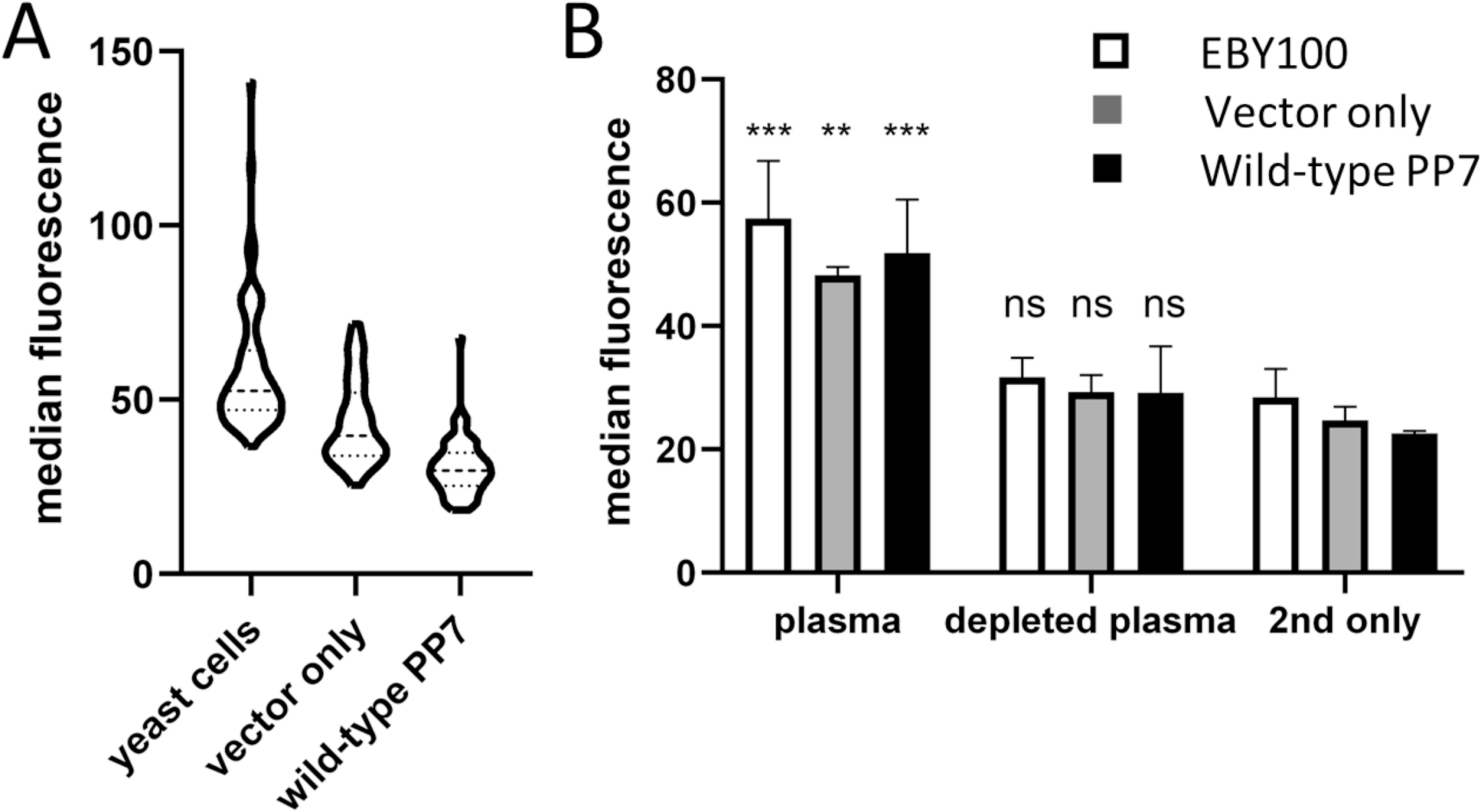
Reducing the potentially high background in plasma samples intended for yeast selections. (A) Reactivity of 50 plasma samples with EBY100 yeast cells and yeast cells transformed with an empty vector or a PP7-expressing construct. (B) Reactivity of a “high background” sample with EBY100 yeast cells and yeast cells transformed with an empty vector or a PP7-expressing construct, before and after depletion of interfering factors with yeast cells. The reaction was measured in duplicates and the comparison with the response to the secondary reagent only was evaluated using one-way ANOVA (ns: not significant, *P* > 0.05, **: 0.01 < *P* < 0.005, ***: *P* < 0.005; 2nd only: secondary reagent only).

## Conclusions

We have developed a yeast display system that allows the efficient presentation of cyclic peptides on the surface of yeast cells, within the scaffold PP7 *Levivirus* capsid protein. After constructing and selecting small-sized libraries of genetically encoding overlapping peptides based on the sequences of the model antigens of choice, we could validate the system by confirming the hits encoding the epitope of the anti-HA and rituximab antibody. Further, we were able to enrich a specific GAD65 epitope-expressing yeast, reactive with polyclonal IgG from plasma with anti-GAD65 reactive antibodies. The system is simple, inexpensive, and highly applicable for defining epitopes of not only monoclonal antibodies but also polyclonal antibodies from complex plasma samples. We believe that this system can in the future be amenable to an expanded range of antigens, for example, by using yeast performing human post-translational modifications. Additionally, its application can be expanded to the high-throughput discovery of antigenic epitopes of clinical importance in diverse autoimmune and allergic conditions. Apart from its prognostic and diagnostic value, this method could deliver antigen-specific treatments that minimize the adverse effects elicited by immunosuppressive therapies currently in use.

## Methods

### Basic Yeast Display Methods

The EBY100 yeast strain (ATCC MYA-4941DQ) was transformed with a pYD1 (vector: RRID:Addgene_73447)-cloned PP7 dimer with dehomologized protomer sequences and selected on an SD-Trp solid medium (1.5% agar, 10 g/L ammonium sulfate, 0.34% yeast nitrogen base, 0.1 M potassium phosphate, pH 6.0, 2% glucose, and 80 mg/L l-leucine). An SD-CAA medium (1% casamino acids, 10 g/L ammonium sulfate, 0.34% yeast nitrogen base, 0.1 M K-phosphate, pH 6.0, 2% glucose) was used for cultivation and an SG/R-CAA medium (2% galactose and 1% raffinose instead of 2% glucose) was used for induction of display clones and libraries. For cultivation, propagation, and storage, standard methods were used.^13,43^ Normalization of display was performed using 66.6 nM anti-V5 antibody (Thermo Fisher Scientific catalog no. MA1–80281, RRID:AB_935873) directed at the C-terminally positioned V5-tag, and in some cases with 6.6 nM anti-Xpress antibody (Thermo Fisher Scientific catalog no. R910–25, RRID:AB_2556552), directed at the N-terminally positioned Xpress-tag. The determination of the copy number of displayed proteins was done using QIFIkit (Agilent) using the anti-Xpress antibody exactly according to the manufacturer’s instructions.

Single inserts and library polymerase chain reaction (PCR) fragments were transformed using PEG-3350/Li-acetate/ssDNA-mediated transformation.^44^ Briefly, EBY100 cultures were expanded in 2 steps in the YPD medium (2% peptone, 1% yeast extract, 2% glucose) at 30 °C on an orbital shaker to an exponential phase, washed once with distilled water, and pretreated with 200 mM Li acetate at 30 °C for 15 min. The transformation mix with 50% PEG-3350, 100 mM Li acetate, 277.8 μg/mL ssDNA, recipient vector, and library components was added and incubated with vigorous shaking at 30 °C. After a heat shock for 45 min at 42 °C, the yeast cells were resuspended in the SD-CAA medium, and aliquots were spread on SD-Trp plates for enumeration. Library cultivation continued for 48 h at 30 °C and was followed with a 1:20 passage for 24 h at 30 °C. The ratio of the insert to the vector was optimized using HA insert and determination of the percentage of the yeast cells staining with the anti-HA antibody (Abcam cat. no. ab130275, RRID:AB_11156884) after transformation.

For the detection of displayed peptides, the yeast cells were cultured on an orbital shaker at 30 °C until the cultures had reached an OD_600_ value of 20–25, and they were induced at an OD_600_ value of 1 for 48 h at 20 °C. Triplicates of induced cultures were measured. For FACS experiments, induced yeast cells were diluted to an OD_600_ value of 1 in 2% BSA-PBS, blocked for 15 min at 20 °C, and stained with antibodies at the dilutions specified below for 1 h at 20 °C. After centrifugation at 1000*g* for 5 min at 20 °C, they were incubated with the secondary reagents for 30 min on ice. An antihuman-Fc-γ-phycoerythrin (PE) conjugate (Sigma-Aldrich catalog no. P8047, RRID:AB_261189) at 1:1000 dilution or an antimouse-Fc-PE conjugate (BioLegend catalog no. 405307, RRID:AB_315010) at 1:500 dilution was used for the detection of binding of antihuman and antimouse primary antibodies, respectively. Normalization was achieved with an anti-V5-tag antibody. Cells were then pelleted at 1000*g* for 5 min at 4 °C and resuspended in 200 μL of ice-cold PBS before the analysis with a Guava flow cytometer (Luminex). All samples were processed in triplicates. Data analysis was performed with Kaluza software, version 2.1. In the used yeast display system, a part of the yeast population (20–30%) is always completely negative for expression, which is why we present the geometric mean of positive cells for well-separated populations after anti-V5 antibody staining and percent positive cells or the median fluorescence intensity or both for other stainings.

### Design of Alternative Cysteine Pairs

The crystal structure (PDB: 2QUD) was analyzed using the DSD-BASE. 44 potential de novo cysteine pairs were proposed and evaluated according to the ability of terminal loop or turn formation, orientation of cysteine side chains, and surface exposure after the visual inspection of the crystal structure. Finally, 2 variants mutated in protomer 1 and 3 variants mutated in protomer 2 were chosen for analysis. Mutagenesis was performed using a Quikchange lightning mutagenesis kit (Agilent) after the introduction of the restriction sites using oligonucleotides listed in Supporting Information Table S2, enabling the gap-repair-driven homologous recombination *in vivo* with PCR fragments produced with oligonucleotides listed in Supporting Information Table S2. For the control, the peptides with additional N- and C-terminal cysteines (designated as “only”) were expressed in the frame with an N-terminal Xpress-tag and a C-terminal V5-tag without a PP7 scaffold.

### Detection of Model Peptides

#### HA-Peptide

For the detection of the HA-tag peptide, the sequence YPYDVPDYAG was inserted at all proposed sites, and the detection antibody was mouse anti-HA clone 16B12, diluted to 6.6 nM.

#### RX-Peptide

The peptide corresponding to the CD20 large extracellular loop (INIYNCEPANPSEKRS; Arg (R) residue deviating from Asn (N) in the native sequence was used due to a better response of this peptide variant) was introduced at all sites encompassed with the *de novo* cysteine pair, and the detection antibody was rituximab (sequence in Supporting Information Table S3) at 4 μM concentration. As the peptide sequence itself contains a single cysteine, we have examined the actual pairing of the cysteine residues by constructing mutations with alanine replacing cysteine 1, 7, or 18 and their combinations, using the QuikChange Lightning site-directed mutagenesis kit with primers listed in Supporting Information Table S2. The detection antibody rituximab was expressed in HEK293–6E cells and purified with standard methods as described before.^45^

#### CX-Meditope Peptide

The 10-meric peptide of the cetuximab meditope sequence (CVFNLGTRRLRC) was inserted at the conventional “MI” site, protomer 1 at site 1 (A1), or devoid of the PP7 scaffold (CX only), and the detection was with cetuximab at 10 μM concentration. The effect of cyclization mediated by cysteines at the MI position was examined with a variant with both cysteines converted to alanine residues, using the QuikChange Lightning site-directed mutagenesis kit with primers listed in Supporting Information Table S2. The detection antibody cetuximab was expressed in HEK293–6E cells and purified with standard methods as described before.^45^

### Peptide Library Construction and Selection

Whole-length sequences of influenza virus hemagglutinin (HA) (GenBank: ACC66770.1), CD20 (GenBank: NM_152866.3), and GAD65 (GenBank: NM_000818.3) were ordered as stretches encoding 15 aa peptides with an overlap of 13 aa or 14 aa as Oligo pools at IDT. The sequences encoded the N- and C-terminal cysteines and had additional 17 nucleotide residues for annealing of the recombination primers allowing the gap-repair-driven homologous recombination in yeast (Supporting Information Table S2). Library inserts were produced using PCR with Q5 HiFi polymerase (New England Biolabs), 0.01 nM of each oligo, and 100 pmol of each recombination primer in a 100 μL reaction. Products were excised from a preparative 2.5% agarose gel, purified using the NucleoSpin DNA extraction kit (Macherey-Nagel), and used for yeast transformation at a 40:1 mass ratio toward the NdeI-linearized acceptor vector with the cloned PP7 dimer sequence. Transformation and selection proceeded as described above. The actual library size was determined by counting the colonies on transformation plates and the quality control was determined by sequencing of about 20 yeast colonies using the PCR product produced with primers pyd1 forward and pyd1 reverse as a template.

For the selection of binding clones, staining with the anti-HA antibody and rituximab in 10% BSA-PBS was performed as described above. Simultaneously with the antihuman IgG conjugate, the anti-V5-tag antibody was added at 66.6 nM for normalization, and double-positive clones were separated in a single round of analysis with a Sony SH8000 sorter and plated out on SD-Trp plates. They were then cultured in 24-well plates in 2 mL of SD-CAA overnight and induced in 2 mL of SG/R-CAA for 48 h at 20 °C. Simultaneous staining with the test antibody and anti-V5-tag antibody was performed, and the samples were analyzed with a Guava flow cytometer. Positive clones were sequenced with the Sanger method using the PCR product produced with primers pyd1 forward and pyd1 reverse. For the peptide sequence plots, the occurrence of individual amino acid residues was multiplied either with the frequency of the individual clone or with the ratio of the specific fluorescence signal (fluorescence of antibody-stained cells minus fluorescence of cells stained with the secondary reagent only) to the fluorescence signal resulting from the anti-V5 antibody used for normalization.

### Testing Plasma Samples for Reactive Peptides and Background Reactivity

Plasma samples of healthy volunteers were obtained from the Austrian Red Cross, from blood samples drawn into EDTA tubes. The anti-GAD65 pretyped plasma was obtained from an FDA-licensed commercial source (PlasmaLab international; Everett, WA 98201) and was processed to obtain the polyclonal antibody preparation using Melon Gel (Thermo Fisher Cat. No. 45206) exactly according to the manufacturer’s instructions. For staining of libraries or single clones, plasma samples or polyclonal antibodies were diluted 1:20 in 10% BSA-PBS, and their binding was detected using the antihuman IgG-PE conjugate. To define the interacting antibody isotype, the detection reagents antihuman kappa-PE (Thermo Fisher Scientific catalog no. MA1–10389, RRID:AB_11151992) or antihuman lambda-PE (Thermo Fisher Scientific catalog no. MA1–10396, RRID:AB_11151966) were used at 1:25 dilution in 2% BSA-PBS to stain the yeast clones displaying most strongly reactive GAD65 peptides. With a plasma sample showing a strong interaction with yeast cells, the reactive component depletion protocol was performed. For this purpose, 4 × 10^8^ EBY100 yeast cells were blocked in 10% BSA-PBS and then incubated in 2 mL of the 1:20 dilution of plasma in 10% BSA-PBS for 1 h at RT with agitation. After a centrifugation step at 1000*g* and 5 min at RT, the supernatant was removed and passed through a 0.45 μm PVDF filter. The depletion of interfering factors was monitored by repeating the staining of yeast cells and detecting the reactivity of the antibody with antihuman IgG-PE.

## Supplementary Material



## Notes

Map of the display vector used for the PP7-mediated peptide display (Figure S1); representative dot plots of FACS analysis of PP7-displayed mutants of the RX-cognate peptide, with the indicated percentage of gated cells and the median fluorescence intensity (Figure S2); phenotypic analysis of the HA-peptide library in the PP7 scaffold displayed on the yeast surface (Figure S3); phenotypic analysis of the CD20 peptide library in the PP7 scaffold displayed on the yeast surface (Figure S4); amino acid and nucleotide sequences of PP7 and protein targets used for display (Table S1); construction methods and oligonucleotide sequences for peptide grafts (Table S2); and amino acid sequences of rituximab and cetuximab, used as model antibodies in this study (Table S3) (PDF)

## §

Austrianni GmbH, Vienna Biocenter 6, Dr.-Bohr-Gasse 7, 1030 Vienna, Austria

## ∥

EFS Unternehmensberatung GmbH, Ungargasse 59–61, 1030 Vienna, Austria

## Abbreviations

aa: amino acid
BSA: bovine serum albumin
CAA: casamino acids
CX: cetuximab
FACS: fluorescence-activated cell sorting
GAD65: glutamic acid decarboxylase 65 kDa isoform
HA: hemagglutinin
PCR: polymerase chain reaction
PBS: phosphate-buffered saline
RT: room temperature
RX: rituximab
