# Supplementary material for: Genetically Encoded Levivirus Coat Protein-Based Yeast Display Libraries of Cyclic Peptides
Source: ACS Synth Biol. 2025 Jul 14;14(8):2987–98. doi: 10.1021/acssynbio.4c00873 (PMC12362601; doi:10.1021/acssynbio.4c00873)
Supplement: Supplementary file 1 [file sb4c00873_si_001.pdf]

Supporting information

Genetically encoded *Levivirus* coat protein-based yeast display libraries of cyclic peptides

Authors

Theodor Simak[1], Florian Stracke[1], Oskar Smrzka[2] and Gordana Wozniak-Knopp[1*]

Affiliations

[1]Christian Doppler Laboratory for Innovative Immunotherapeutics, BOKU University, Muthgasse 18, 1190 Vienna, Austria;

present address: T.S.: Austrianni GmbH, Vienna Biocenter 6, Dr.-Bohr-Gasse 7, 1030 Vienna, Austria, F.S.: EFS Unternehmensberatung GmbH, Ungargasse 59-61, 1030 Vienna, Austria

[2]Ablevia biotech GmbH, Maria Jacobi Gasse 1, 1030 Vienna, Austria

[*]Correspondence should be addressed to: G. Wozniak Knopp (gordana.wozniak@boku.ac.at)

Supplementary Figures

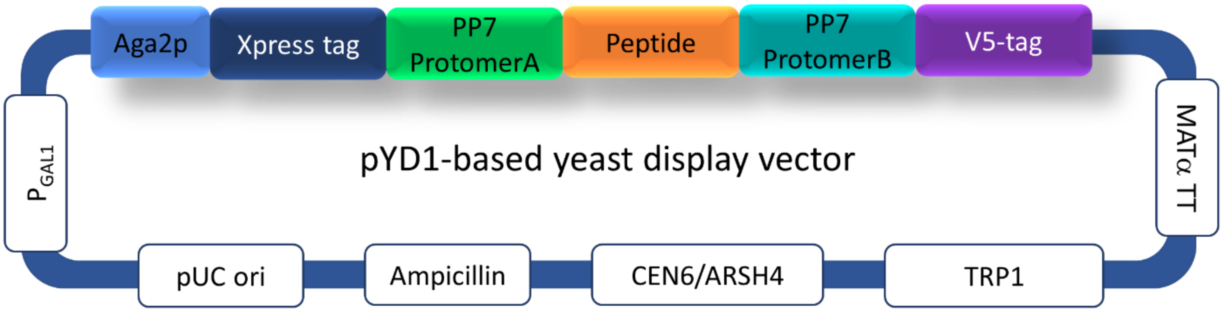

Figure S1. Map of the display vector used for the PP7-mediated peptide display. Elements of the expression cassette: Aga2p, agglutinin Aga2p expression construct; Xpress tag; Protomer A of PP7 capsid protein; inserted peptide; Protomer B of PP7 capsid protein; V5-tag. Other elements: P_{GAL1}, *GAL1* promotor; MATαTT, MATα transcription termination region; pUC ori, pUC origin of replication; Ampicillin, ampicillin resistance gene open reading frame; CEN6/ARSH4, Chromosome VI centromere/Autonomously Replicating Sequence 4 cassette; TRP1, *TRP1* gene open reading frame.

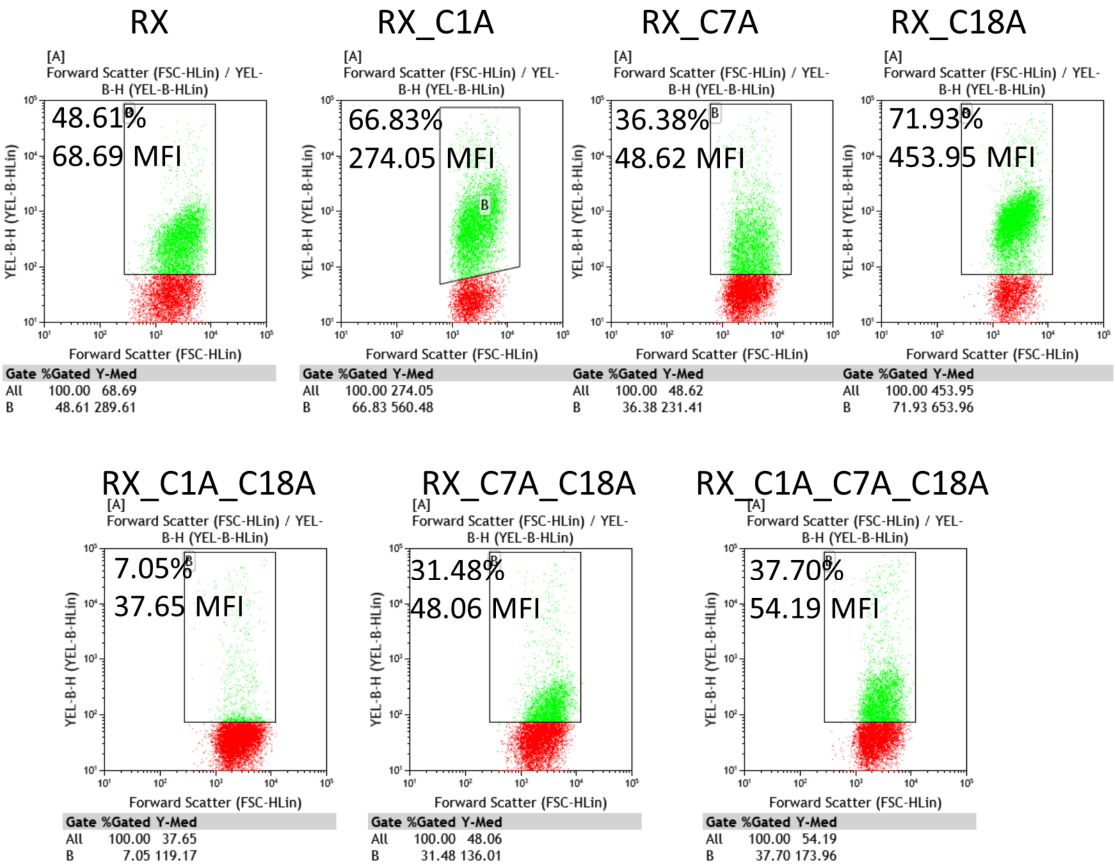

Figure S2. Representative dot plots of FACS analysis of PP7-displayed mutants of RX-cognate peptide, with indicated percentage of gated cells and median fluorescence intensity. Gates were set based on the cells stained with secondary reagent only.

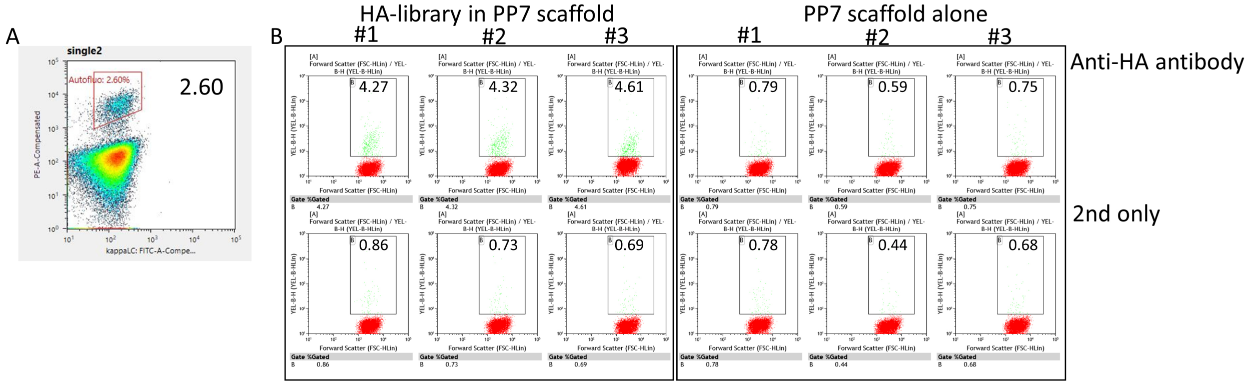

Figure S3. Phenotypic analysis of HA-peptide library in PP7 scaffold displayed on yeast surface. (A) Dot plot of the library cells stained with the anti-HA antibody before sorting, with indicated percentage of cells to-be-captured. (B) Representative dot plots of 3 parallel yeast display cultures of the HA-library in PP7 scaffold and the scaffold alone, stained with anti-HA antibody or secondary reagent only (2nd only), with indicated percentage of positive cells. Gates were set based on the cells stained with secondary reagent only.

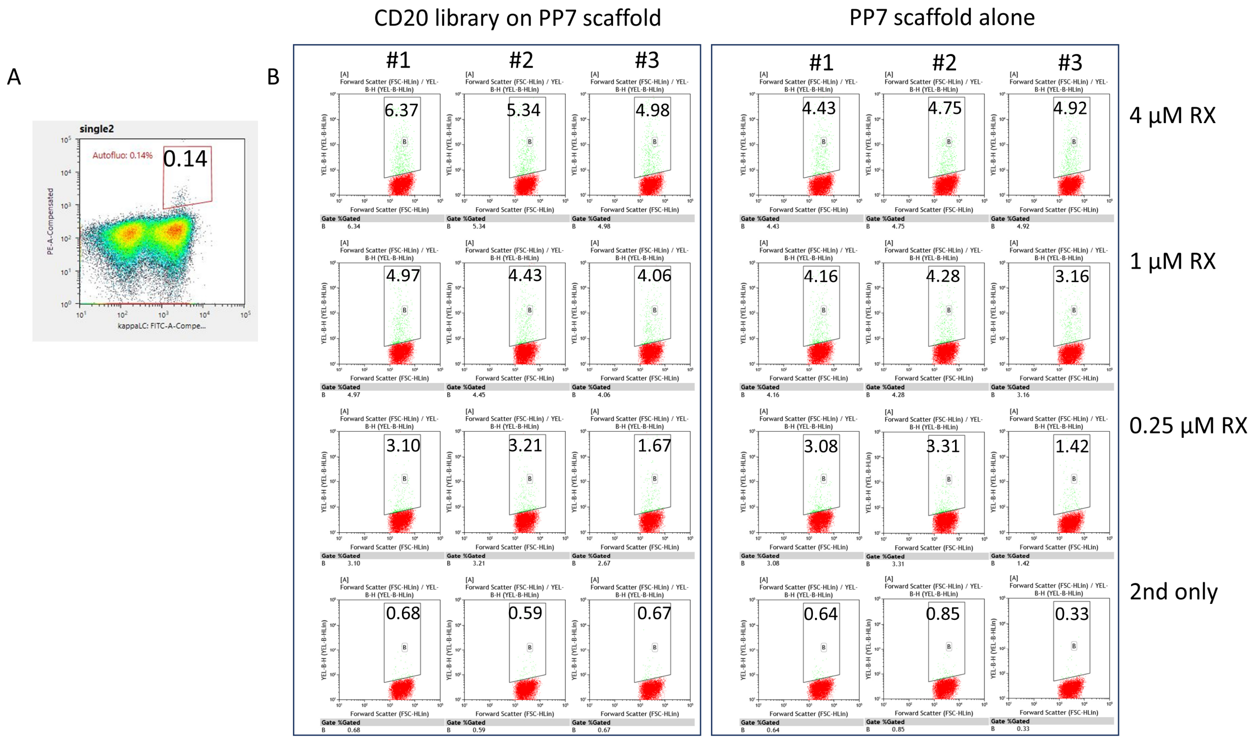

Figure S4. Phenotypic analysis of CD20 peptide library in PP7 scaffold displayed on yeast surface. (A) Dot plot of the library cells stained with the RX antibody before sorting, with indicated percentage of cells to-be-captured. (B) Representative dot plots of 3 parallel yeast display cultures of the CD20 library in PP7 scaffold and the scaffold alone, stained with 4, 1, or 0.25 µM RX antibody or secondary reagent only (2nd only). Gates were set based on the cells stained with secondary reagent only.

Table S1. Amino acid- and nucleotide sequences of PP7, peptides, and protein targets used for display

PP7 capside protein

Protein sequence

SKTIVLSVGEATRTLTEIQSTADRQIFEEKVGPLVGRLRLTASLRQNGAKTAYRVNLKLDQADVVDCSTSVCGELPKVRYTQVWSHDVTIVANST
EASRKSLYDLTKSLVATSQVEDLVVNLVPLGRAYGGSKTIVLSVGEATRTLTEIQSTADRQIFEEKVGPLVGRLRLTASLRQNGAKTAYRVNLKL
DQADVVDCSTSVCGELPKVRYTQVWSHDVTIVANSTEASRKSLYDLTKSLVATSQVEDLVVNLVPLGR

Nucleotide sequence, NdeI site underlined

AGCAAGACCATCGTACTGAGCGTGGGCGAGGCCACCAGGACCCTGACCGAGATCCAGAGCACCGCCGACAGGCAGATCTTCGAGGAGAAGGTGGG
CCCCCTGGTGGGCAGGCTGAGGCTGACCGCCAGCCTGAGGCAGAACGGCGCCAAGACCGCCTACAGGGTGAACCTGAAGCTGGACCAGGCCGACG
TGGTGGACTGCAGCACCAGCGTGTGCGGCGAGCTGCCCAAGGTGAGGTACACCCAGGTGTGGAGCCACGACGTGACCATCGTGGCCAACAGCACC
GAGGCCAGCAGGAAGAGCCTGTACGACCTGACCAAGAGCCTGGTGGCCACCAGCCAGGTGGAGGACCTGGTGGTGAATCTAGTCCCTCTTGGAAG
AG<u>CATATG</u>GCGGCTCTAAAACTATTGTTTTGTCTGTTGGTGAAGCTACTAGAACTTTGACTGAAATTCAATCTACTGCTGATAGACAAATTTTTG
AAGAAAAAGTTGGTCCATTGGTTGGTAGATTGAGATTGACTGCTTCTTTGAGACAAAATGGTGCTAAAACTGCTTATAGAGTTAATTTGAAATTG
GATCAAGCTGATGTTGTTGATTGTTCTACTTCTGTTTGTGGTGAATTGCCAAAAGTTAGATATACTCAAGTTTGGTCTCATGATGTTACTATTGT
TGCTAATTCTACTGAAGCTTCTAGAAAATCTTTGTATGATTTGACTAAATCTTTGGTTGCTACTTCTCAAGTTGAAGATTTGGTTGTTAATTTGG
TTCCATTGGGTAGA

Peptides and proteins for PP7-mediated display

HA

YPYDVPDYAG

RX

CINIYNCEPANPSEKRSC

CX

CVFNLGTRRLRC

Influenza hemagglutinin

Accession number	ACC66770.1
Number of fragments	155

Protein sequence

QKLPGNDNSTATLCLGHHAVPNGTIVKTITNDQIEVTNATELVQSSSTGGICDSPHQILDGENCTLIDALLGDPQCDGFQNKKWDLFVERSKAHS
NCYPYDVPDYASLRSLIASSGTLEFNNESFNWTGVTQNGTSSSCKRRSNQSFFSRLNWLTHLKFKYPALNVTMPNNEKFDKLYIWGVHHPGTDSD
QISLYAQAPGRITVSTKRSQQTVIPNIGFRPRVRDISSRISIYWTIVKPGDILLINSTGNLIAPRGYFKIRSGKSSIMKSDAPIGKCNSECITPN
GSIPNDKPFQNVNRITYGACPRYVKQNTLKLATGMRNVPEKQTR

Nucleotide sequence

CAAAAACTTCCCGGAAATGACAACAGCACGGCAACGCTGTGCCTTGGGCACCATGCAGTACCAAACGGAACGATAGTGAAAACAATCACGAATGA
CCAAATTGAAGTTACTAATGCTACTGAGCTGGTTCAGAGTTCCTCAACAGGTGGAATATGCGACAGCCCTCATCAGATCCTTGATGGAGAAAACT
GCACACTAATAGATGCTCTATTGGGAGACCCTCAGTGTGATGGCTTCCAAAATAAGAAATGGGACCTTTTTGTTGAACGCAGCAAAGCCCACAGC
AACTGTTACCCTTATGATGTGCCGGATTATGCCTCCCTTAGGTCACTAATTGCCTCATCCGGCACACTGGAGTTTAACAATGAAAGCTTCAATTG
GACTGGAGTCACTCAGAATGGAACAAGCTCTTCTTGCAAAAGGAGATCTAATCAAAGTTTCTTTAGTAGATTGAATTGGTTGACCCATTTAAAAT
TCAAATACCCAGCATTGAACGTGACTATGCCAAACAATGAAAAATTTGACAAATTGTACATTTGGGGGGTTCACCACCCGGGTACGGACAGTGAC
CAAATCAGCCTATATGCTCAAGCACCAGGAAGAATCACAGTCTCTACCAAAAGAAGCCAACAAACTGTAATCCCGAATATCGGATTTAGACCCAG
GGTAAGGGATATCTCCAGCAGAATAAGCATCTATTGGACAATAGTAAAACCGGGAGACATACTTTTGATTAACAGCACAGGGAATCTAATTGCTC
CTCGGGGTTACTTCAAAATACGAAGTGGGAAAAGCTCAATAATGAAATCAGATGCACCCATTGGCAAATGCAATTCTGAATGCATCACTCCAAAT
GGAAGCATTCCCAATGACAAACCATTTCAAAATGTAAACAGAATCACATATGGGGCCTGTCCCAGATATGTTAAGCAAAACACTCTGAAATTGGC
AACAGGGATGCGAAATGTACCAGAGAAACAAACTAGA

CD20

Accession number	NCBI NM_152866.3
Number of fragments	139

Protein sequence

MTTPRNSVNGTFPAEPMKGPIAMQSGPKPLFRRMSSLVGPTQSFFMRESKTLGAVQIMNGLFHIALGGLLMIPAGIYAPICVTVWYPLWGGIMYI
ISGSLLAATEKNSRKCLVKGKMIMNSLSLFAAISGMILSIMDILNIKISHFLKMESLNFIRAHTPYINIYNCEPANPSEKNSPSTQYCYSIQSLF
LGILSVMLIFAFFQELVIAGIVENEWKRTCSRPKSNIVLLSAEEKKEQTIEIKEEVVGLTETSSQPKNEEDIEIIPIQEEEEEETETNFPEPPQD
QESSPIENDSSP

Nucleotide sequence

ATGACAACACCCAGAAATTCAGTAAATGGGACTTTCCCGGCAGAGCCAATGAAAGGCCCTATTGCTATGCAATCTGGTCCAAAACCACTCTTCAG
GAGGATGTCTTCACTGGTGGGCCCCACGCAAAGCTTCTTCATGAGGGAATCTAAGACTTTGGGGGCTGTCCAGATTATGAATGGGCTCTTCCACA
TTGCCCTGGGGGGTCTTCTGATGATCCCAGCAGGGATCTATGCACCCATCTGTGTGACTGTGTGGTACCCTCTCTGGGGAGGCATTATGTATATT
ATTTCCGGATCACTCCTGGCAGCAACGGAGAAAAACTCCAGGAAGTGTTTGGTCAAAGGAAAAATGATAATGAATTCATTGAGCCTCTTTGCTGC
CATTTCTGGAATGATTCTTTCAATCATGGACATACTTAATATTAAAATTTCCCATTTTTTAAAAATGGAGAGTCTGAATTTTATTAGAGCTCACA
CACCATATATTAACATATACAACTGTGAACCAGCTAATCCCTCTGAGAAAAACTCCCCATCTACCCAATACTGTTACAGCATACAATCTCTGTTC
TTGGGCATTTTGTCAGTGATGCTGATCTTTGCCTTCTTCCAGGAACTTGTAATAGCTGGCATCGTTGAGAATGAATGGAAAAGAACGTGCTCCAG
ACCCAAATCTAACATAGTTCTCCTGTCAGCAGAAGAAAAAAAAGAACAGACTATTGAAATAAAAGAAGAAGTGGTTGGGCTAACTGAAACATCTT

CCCAACCAAAGAATGAAGAAGACATTGAAATTATTCCAATCCAAGAAGAGGAAGAAGAAGAAACAGAGACGAACTTTCCAGAACCTCCCCAAGAT
CAGGAATCCTCACCAATAGAAAATGACAGCTCTCCT

GAD65

Accession number NM_001134366.2

Number of fragments 286

Protein sequence

MASPGSGFWSFGSEDGSGDSENPGTARAWCQVAQKFTGGIGNKLCALLYGDAEKPAESGGSQPPRAAARKAACACDQKPCSCSKVDVNYAFLHAT
DLLPACDGERPTLAFLQDVMNILLQYVVKSFDRSTKVIDFHYPNELLQEYNWELADQPQNLEEILMHCQTTLKYAIKTGHPRYFNQLSTGLDMVG
LAADWLTSTANTNMFTYEIAPVFVLLEYVTLKKMREIIGWPGGSGDGIFSPGGAISNMYAMMIARFKMFPEVKEKGMAALPRLIAFTSEHSHFSL
KKGAAALGIGTDSVILIKCDERGKMIPSDLERRILEAKQKGFVPFLVSATAGTTVYGAFDPLLAVADICKKYKIWMHVDAAWGGGLLMSRKHKWK
LSGVERANSVTWNPHKMMGVPLQCSALLVREEGLMQNCNQMHASYLFQQDKHYDLSYDTGDKALQCGRHVDVFKLWLMWRAKGTTGFEAHVDKCL
ELAEYLYNIIKNREGYEMVFDGKPQHTNVCFWYIPPSLRTLEDNEERMSRLSKVAPVIKARMMEYGTTMVSYQPLGDKVNFFRMVISNPAATHQD
IDFLIEEIERLGQDL

Nucleotide sequence

ATGGCATCTCCGGGCTCTGGCTTTTGGTCTTTCGGGTCGGAAGATGGCTCTGGGGATTCCGAGAATCCCGGCACAGCGCGAGCCTGGTGCCAAGT
GGCTCAGAAGTTCACGGGCGGCATCGGAAACAAACTGTGCGCCCTGCTCTACGGAGACGCCGAGAAGCCGGCGGAGAGCGGCGGGAGCCAACCCC
CGCGGGCCGCCGCCCGGAAGGCCGCCTGCGCCTGCGACCAGAAGCCCTGCAGCTGCTCCAAAGTGGATGTCAACTACGCGTTTCTCCATGCAACA
GACCTGCTGCCGGCGTGTGATGGAGAAAGGCCCACTTTGGCGTTTCTGCAAGATGTTATGAACATTTTACTTCAGTATGTGGTGAAAAGTTTCGA
TAGATCAACCAAAGTGATTGATTTCCATTATCCTAATGAGCTTCTCCAAGAATATAATTGGGAATTGGCAGACCAACCACAAAATTTGGAGGAAA
TTTTGATGCATTGCCAAACAACTCTAAAATATGCAATTAAAACAGGGCATCCTAGATACTTCAATCAACTTTCTACTGGTTTGGATATGGTTGGA
TTAGCAGCAGACTGGCTGACATCAACAGCAAATACTAACATGTTCACCTATGAAATTGCTCCAGTATTTGTGCTTTTGGAATATGTCACACTAAA
GAAAATGAGAGAAATCATTGGCTGGCCAGGGGGCTCTGGCGATGGGATATTTTCTCCCGGTGGCGCCATATCTAACATGTATGCCATGATGATCG
CACGCTTTAAGATGTTCCCAGAAGTCAAGGAGAAAGGAATGGCTGCTCTTCCCAGGCTCATTGCCTTCACGTCTGAACATAGTCATTTTTCTCTC
AAGAAGGGAGCTGCAGCCTTAGGGATTGGAACAGACAGCGTGATTCTGATTAAATGTGATGAGAGAGGGAAAATGATTCCATCTGATCTTGAAAG
AAGGATTCTTGAAGCCAAACAGAAAGGGTTTGTTCCTTTCCTCGTGAGTGCCACAGCTGGAACCACCGTGTACGGAGCATTTGACCCCCTCTTAG
CTGTCGCTGACATTTGCAAAAAGTATAAGATCTGGATGCATGTGGATGCAGCTTGGGGTGGGGGATTACTGATGTCCCGAAAACACAAGTGGAAA
CTGAGTGGCGTGGAGAGGGCCAACTCTGTGACGTGGAATCCACACAAGATGATGGGAGTCCCTTTGCAGTGCTCTGCTCTCCTGGTTAGAGAAGA
GGGATTGATGCAGAATTGCAACCAAATGCATGCCTCCTACCTCTTTCAGCAAGATAAACATTATGACCTGTCCTATGACACTGGAGACAAGGCCT
TACAGTGCGGACGCCACGTTGATGTTTTTAAACTATGGCTGATGTGGAGGGCAAAGGGGACTACCGGGTTTGAAGCGCATGTTGATAAATGTTTG
GAGTTGGCAGAGTATTTATACAACATCATAAAAAACCGAGAAGGATATGAGATGGTGTTTGATGGGAAGCCTCAGCACACAAATGTCTGCTTCTG
GTACATTCCTCCAAGCTTGCGTACTCTGGAAGACAATGAAGAGAGAATGAGTCGCCTCTCGAAGGTGGCTCCAGTGATTAAAGCCAGAATGATGG
AGTATGGAACCACAATGGTCAGCTACCAACCCTTGGGAGACAAGGTCAATTTCTTCCGCATGGTCATCTCAAACCCAGCGGCAACTCACCAAGAC
ATTGACTTCCTGATTGAAGAAATAGAACGCCTTGGACAAGATTTA

Table S2. Construction methods and oligonucleotide sequences for peptide grafts

**Peptide grafts**	Construction method and oligonucleotides	Oligonucleotide sequences
**Position: MI (between protomers)**		
Recipient vector	Linearize with *Nde*I	
HA-tag	PCR and recombination	
	pp7_hatag_recfor	GTGAATCTAGTCCCTCTTGGAAGAGCATATTAC
	pp7_hatag_recrev	GACAAAACAATAGTTTTAGAGCCGCCGCCTGC
CX-meditope	PCR and recombination	
	pp7_CXmi_recfor	GTGAATCTAGTCCCTCTTGGAAGAGCATATTGTG
	pp7_CXmi_recrev	GACAAAACAATAGTTTTAGAGCCGCCACATCTG
RX-peptide	PCR and recombination	
	pp7_RXp1mi_recfor	GTGGTGAATCTAGTCCCTCTTGGAAGAGCATATTGTATTAACATATACAAC
	pp7_RXp1mi_recrev	GACAAAACAATAGTTTTAGAGCCGCCGCAGCTCCTTTTCTCAGAGGG
**Position: A1 (protomer 1, mutant 1)**		
Recipient vector	Mutagenesis to introduce BsiWI site, linearize with BsiWI	
	pp7_bsiw1	GTGGGCGAGGCCACCCGTACGCTGACCGAGATCCAG
	pp7_bsiw1a	CTGGATCTCGGTCAGCGTACGGGTGGCCTCGCCCAC
HA-tag	PCR and recombination	
	pp7_hatag_p11_recfor	GGTACCAGGATCCAGCAAGACCATCGTACTGAGCTACCCTTATGACGTACCA G
	pp7_hatag_p11_recrev	GCGGTGCTCTGGATCTCGGTCAGCGTACGGGTGCCTGCGTAGTCTGGTAC
CX-meditope	PCR and recombination	
	pp7_cx_p11_recfor	ATCCAGCAAGACCATCGTACTGAGCTGTGTGTTCAACCTAGG
	pp7_cx_p11_recrev	CGGCGGTGCTCTGGATCTCGGTCAGCGTACGGGTACATCTGAGCCGGCGAG
RX-peptide	PCR and recombination	
	pp7_rxp1_p11_recfor	ATCCAGCAAGACCATCGTACTGAGCTGTATTAACATATACAAC
	pp7_rxp1_p11_recrev	CGGCGGTGCTCTGGATCTCGGTCAGCGTACGGGTGCAGCTCCTTTTCTCAGA GGG
**Position: A2 (protomer 1, mutant 2)**		
Recipient vector	Linearize with BglII and Bpu10I	
HA-tag	PCR and recombination	
	pp7_hatag_p12_recfor	AGATCCAGAGCACCGCCGACAGGCAGATCTTCGAGTACCCTTATGACGTACC AG
	pp7_hatag_p12_recrev	CCGTTCTGTCGTAAACTGGCGGTCAGCCTACCTGCGTAGTCTGGTAC
RX-peptide	PCR and recombination	
	pp7_rxp1_p12_recfor	AGATCCAGAGCACCGCCGACAGGCAGATCTTCGAGTGTATTAACATATACAA C
	pp7_rxp1_p12_recrev	CCGTTCTGCCTCAGGCTGGCGGTCAGCCTACAGCTCCTTTTCTCAGAGGG
**Position: B1 (protomer 2, mutant 1)**		
Recipient vector	Mutagenesis to introduce BsiWI site , linearize with BsiWI	
	pp7_bsiw2	GTTGGTGAAGCTACTCGTACGTTGACTGAAATTCAATC
	pp7_bsiw2a	GATTGAATTTCAGTCAACGTACGAGTAGCTTCACCAAC
HA-tag	PCR and recombination	
	pp7_hatag_p21_recfor	CGGCTCTAAAACTATTGTATTGTCTTACCCTTATGACGTACCAG
	pp7_hatag_p21_recrev	CAGCAGTAGATTGAATTTCAGTCAACGTACGAGTGCCTGCATAGTCTGGTAC
RX-peptide	PCR and recombination	
	pp7_rxp1_p21_recfor	CGGCTCTAAAACTATTGTTTTGTCTTGCATTAACATATACAAC

	pp7_rxp1_p21_recrev	CAGCAGTAGATTGAATTTCAGTCAACGTACGAGTACAGCTCCTTTTCTCAGA GGG
**Position: B2 (protomer 2, mutant 2)**		
Recipient vector	Linearize with PflMI	
HA-tag	PCR and recombination	
	pp7_hatag_p22_recfor	TACTGCTGATAGACAAATTTTTGAATACCCTTATGACGTACCAG
	pp7_hatag_p22_recrev	TTTGTCTCAAAGAAGCTGTCAATCTGCCTGCGTAGTCTGGTAC
RX-peptide	PCR and recombination	
	pp7_rxp1_p22_recfor	TACTGCTGATAGACAAATTTTTGAATGTATTAACATATACAAC
	pp7_rxp1_p22_recrev	TTTGTCTCAAAGAAGCAGTCAATCTACAGCTCCTTTTCTCAGAGGG
**Position: B4 (protomer 2, mutant 4):**		
Recipient vector	Mutagenesis to introduce EcoRI site, linearize with EcoRI	
	pp7_ecor2	TGTTACTATTGTTGCGAATTCTACTGAAGCT
	pp7_ecor2a	AGCTTCAGTAGAATTCGCAACAATAGTAACA
HA-tag	PCR and recombination	
	pp7_hatag_p24_recfor	AGTTTGGTCTCATGATGTTACTATTTACCCTTATGACGTACCAG
	pp7_hatag_p24_recrev	ACAAAGATTTTCTAGAAGCTTCAGTGCCTGCGTAGTCTGGTAC
RX-peptide	PCR and recombination	
	pp7_rxp1_p24_recfor	AGTTTGGTCTCATGATGTTACTATTTGTATTAACATATACAAC
	pp7_rxp1_p24_recrev	ACAAAGATTTTCTAGAAGCTTCAGTGCAGCTCCTTTTCTCAGAGGG
**Position: Only (pYD1, no PP7)**		
HA-tag	PCR and recombination	
	HAonly_recfor	GACGATGACGATAAAGTACCAGGATCCTACCCGTACGACGTAC
	HAonly_recrev	GGGACCTCTAGACTCGAGCGGCCGTGCACCTGCATAGTCTGG
CX-meditope	PCR and recombination	
	pyd_cx_recfor	GTACGACGATGACGATAAGGTACCAGGATCCTGCGTGTTCAACCTAGG
	pyd_cx_recrev	CTCGCCGGCTCAGATGCGCGCGGCCGCTCGAGTCTAGAGGGCCCTTCGAAGG
RX-peptide	PCR and recombination	
	pyd_rxp1_recfor	GTACGACGATGACGATAAGGTACCAGGATCCTGTATTAACATATACAAC
	pyd_rxp1_recrev	CCTTCGAAGGGCCCTCTAGACTCGAGCGGCCGCGCACAGCTCCTTTTCTCAG AGGG
**Alanine mutagenesis**		
	Site-directed mutagenesis	
**CX-meditope**		
**CX_C1A**	cx_mi_c1a	CTTGGAAGAGCATATGCAGTGTTCAACCTAGGA
	cx_mi_c1aa	TCCTAGGTTGAACACTGCATATGCTCTTCCAAG
**CX_C12A**	cx_mi_c12a	ACTCGACGGCTCAGGGCTGGCGGCTCTAAAACT
	cx_mi_c12aa	AGTTTTAGAGCCGCCAGCCCTGAGCCGTCGAGT
**RX-peptide**		
**RX_C1A**	rxp1_c1a	CTTGGAAGAGCATATGCAATTAACATATACAAC
	rxp1_c1aa	GTTGTATATGTTAATTGCATATGCTCTTCCAAG
**RX_C7A**	rxp1_c7a	GTATTAACATATACAACGCAGAACCAGCTAATCCC
	rxp1_c7aa	GGGATTAGCTGGTTCTGCGTTGTATATGTTAATAC
**RX_C18A**	rxp1_c18a	TCTGAGAAAAGGAGCGCGGGCGGCTCTAAAACT
	rxp1_c18aa	AGTTTTAGAGCCGCCCGCGCTCCTTTTCTCAGA
**Library cloning**	PCR and recombination	
	pp7lib_for_long	GTGGTGAATCTAGTCCCTCTTGGAAGAGCATATTGT
	pp7lib_rev_long	GACAAAACAATAGTTTTAGAGCCGCCGCA

**Sequencing primers**	pyd1 forward	AGTAACGTTTGTCAGTAATTGC
	pyd1 reverse	GTCGATTTTGTTACATCTACAC

Table S3. Amino acid sequences of rituximab and cetuximab, used as model antibodies in this study

Rituximab
Heavy chain
QVQLQQPGAELVKPGASVKMSCKASGYTFTSYNMHWVKQTPGRGLEWIGAIYPGNGDTSYNQKFKGKATLTADKSS<br>STAYMQLSSLTSEDSAVYYCARSTYYGGDWYFNVWGAGTTVTVSAASTKGPSVFPLAPSSKSTSGGTAALGCLVKD<br>YFPEPVTVSWNSGALTSGVHTFPAVLQSSGLYSLSSVVTVPSSSLGTQTYICNVNHKPSNTKVDKKVEPKSCDKTH<br>TCPPCPAPELLGGPSVFLFPPKPKDTLMISRTPEVTCVVVDVSHEDPEVKFNWYVDGVEVHNAKTKPREEQYNSTY<br>RVVSVLTVLHQDWLNGKEYKCKVSNKALPAPIEKTISKAKGQPREPQVYTLPPSRDELTKNQVSLTCLVKGFYPSD<br>IAVEWESNGQPENNYKTTPPVLDSDGSFFLYSKLTVDKSRWQQGNVFSCSVMHEALHNHYTQKSLSLSPGK
Light chain
QIVLSQSPAILSASPGEKVTMTCRASSSVSYIHWFQQKPGSSPKPWIYATSNLASGVPVRFSGSGSGTSYSLTISR<br>VEAEDAATYYCQQWTSNPPTFGGGTKLEIKRTVAAPSVFIFPPSDEQLKSGTASVVCLLNNFYPREAKVQWKVDNA<br>LQSGNSQESVTEQDSKDSTYSLSSTLTLSKADYEKHKVYACEVTHQGLSSPVTKSFNRGEC
Cetuximab
Heavy chain
QVQLKQSGPGLVQPSQSLSITCTVSGFSLTNYGVHWVRQSPGKGLEWLGVIWSGGNTDYNTPFTSRLSINKDNSKS<br>QVFFKMNSLQSNDTAIYYCARALTYYDYEFAYWGQGTLVTVSAASTKGPSVFPLAPSSKSTSGGTAALGCLVKDYF<br>PEPVTVSWNSGALTSGVHTFPAVLQSSGLYSLSSVVTVPSSSLGTQTYICNVNHKPSNTKVDKKVEPKSCDKTHTC<br>PPCPAPELLGGPSVFLFPPKPKDTLMISRTPEVTCVVVDVSHEDPEVKFNWYVDGVEVHNAKTKPREEQYNSTYRV<br>VSVLTVLHQDWLNGKEYKCKVSNKALPAPIEKTISKAKGQPREPQVYTLPPSRDELTKNQVSLTCLVKGFYPSDIA<br>VEWESNGQPENNYKTTPPVLDSDGSFFLYSKLTVDKSRWQQGNVFSCSVMHEALHNHYTQKSLSLSPGK
Light chain
DILLTQSPVILSVSPGERVSFSCRASQSIGTNIHWYQQRTNGSPRLLIKYASESISGIPSRFSGSGSGTDFTLSIN<br>SVESEDIADYYCQQNNNWPTTFGAGTKLELKRTVAAPSVFIFPPSDEQLKSGTASVVCLLNNFYPREAKVQWKVDN<br>ALQSGNSQESVTEQDSKDSTYSLSSTLTLSKADYEKHKVYACEVTHQGLSSPVTKSFNRGEC
